# Synthesis and Characterization of Biodegradable Terpolymer Scale Inhibitors

**DOI:** 10.3390/ma18174163

**Published:** 2025-09-05

**Authors:** Fei Gao, Peng Xu, Yongqing Zhang, Hui Zhang

**Affiliations:** 1 School of Petroleum Engineering, Yangtze University, Wuhan 430100, China; 501092@yangtzeu.edu.cn; 2 National Engineering Research Center for Oil & Gas Drilling and Completion Technology, Yangtze University, Wuhan 430100, China; 3 Hubei Key Laboratory of Oil and Gas Drilling and Production Engineering, Yangtze University, Wuhan 430100, China; 4 State Key Laboratory of Low Carbon Catalysis and Carbon Dioxide Utilization, Yangtze University, Wuhan 430100, China; 5 Sinopec North China Company, Zhengzhou 450006, China; zhangyqing.hbsj@sinopec.com (Y.Z.); gczhanghui.hbsj@sinopec.com (H.Z.)

**Keywords:** scale inhibitor, terpolymer, calcium carbonate scale, calcium sulfate scale, biodegradable

## Abstract

To address scaling issues in oilfield water injection, a degradable terpolymer scale inhibitor MA-AA-AMPS (terpolymer)was synthesized via aqueous solution polymerization using maleic anhydride, acrylic acid, and 2-acrylamido-2-methylpropanesulfonic acid as monomers. Characterization confirmed the presence of carboxyl, sulfonic, and amide groups in the copolymer with good thermal stability. Scale inhibition tests showed that at 2% dosage, its scale inhibition efficiency exceeded 80%, remaining above 80% in the pH range of 3–8 and over 50% at 150 °C, with excellent tolerance to high-calcium environments. Biodegradation tests revealed BOD_5_/COD > 0.3, with a biodegradation rate exceeding 50% in 15 days and reaching 83.4% in 30 days, indicating environmental friendliness. This scale inhibitor effectively solves scaling problems in oilfield water injection systems.

## 1. Introduction

With the continuous development of reservoirs, formation pressure gradually decreases. Water injection recovery, as an important means to enhance oil recovery, can supplement energy to the formation, thereby maintaining formation pressure and improving reservoir recovery efficiency [1,2]. In the process of oil exploitation, produced water is usually treated and reinjected to improve the recycling rate of water. However, oilfield wastewater generally contains a large amount of impurities, such as salt ions and suspended solid particles. Affected by environmental factors such as temperature, pH, and pressure, these impurities easily lead to the formation of scale in water injection pipelines, which seriously affects the implementation of water injection technology [3]. During oilfield production, the content of calcium ions, carbonates, and sulfates in water is very high, resulting in the formation of a large amount of CaCO_3_ and CaSO_4_ scales [4,5,6,7,8].

To address the scaling issue in water injection pipelines, experts and scholars both domestically and internationally have developed a series of scale inhibitors [9,10]. Currently, commonly used scale inhibitors are mainly classified into natural polymer-based [11,12], inorganic polyphosphate-based [13,14], organic phosphate-based [15], and polymer-based scale inhibitors [16,17,18,19]. Natural polymer scale inhibitors exhibit excellent degradability and low cost, but they require large dosages and have relatively poor scale inhibition efficiency. Inorganic polyphosphate scale inhibitors are prone to causing environmental pollution, leading to eutrophication of water bodies, and they are also susceptible to hydrolysis, failing to meet the needs of long-term water injection production in oilfields [20]. Organic phosphate scale inhibitors possess characteristics such as temperature resistance and acid-alkali resistance, but they also face the environmental risk of phosphorus pollution [21]. Polymer scale inhibitors have relatively good scale inhibition effects, but they still suffer from technical challenges, such as insufficient temperature resistance and difficulty in degradation [22].

To address the technical bottlenecks of the aforementioned scale inhibitors, terpolymer scale inhibitors have become a research focus in oilfield water injection scale inhibition in recent years, as their functional groups can be regulated via monomer combination. Their core advantage stems from the synergistic effect of carboxyl groups (-COOH) and sulfonic acid groups (-SO_3_H). Carboxyl groups are key scale-inhibiting moieties, exerting effects by chelating Ca^2+^ and disrupting the lattices of CaCO_3_/CaSO_4_. For instance, p(IA-ANA-ACMO) prepared by Chen et al. [23] achieves static scale inhibition rates of 97.7% and 98.1% against CaCO_3_ and CaSO_4_, respectively, and a dynamic rate of 96.6% at a dosage of 30 mg/L, confirming the supporting role of carboxyl groups. Sulfonic acid groups, on the other hand, mitigate the defect of carboxyl groups (prone to calcium gel formation); their strong hydrophilicity, salt resistance, and temperature resistance enhance adaptability to complex water conditions. As exemplified by IA-PEG-SAMS developed by Xiao et al. [24], under optimal conditions, it reaches scale inhibition rates of 95.16% (for CaCO_3_ at 90 mg/L) and 92.13% (for CaSO_4_ at 0.2 mg/L). However, current research still faces challenges. Firstly, in molecular design, it is difficult to precisely regulate the ratio, distribution, and interaction strength of carboxyl and sulfonic acid groups, and the micro-mechanism of their synergistic scale inhibition remains incompletely clarified. Secondly, in application adaptability, most existing copolymers are designed for single operating conditions; when exposed to oilfield environments with high salinity, wide temperature ranges, and fluctuating pH, their scale inhibition stability and long-term effectiveness are insufficient [25]. Additionally, some copolymers exhibit poor degradability and inadequate compatibility with formation fluids, making it hard to balance the requirements of high efficiency, environmental friendliness, and on-site adaptability [22,26].

Based on this, this study focuses on the scaling in oilfield water injection systems. Using maleic anhydride, acrylic acid, and 2-acrylamido-2-methylpropanesulfonic acid as monomers, a new biodegradable terpolymer scale inhibitor, MA-AA-AMPS, was prepared via aqueous solution polymerization. Systematic studies were conducted on its molecular structure characterization, scale inhibition performance testing, thermal stability analysis, and biodegradability evaluation, with comparisons made against commonly used phosphoric acid scale inhibitors. The aim is to reveal its scale inhibition mechanism and application potential, thereby providing a theoretical basis and technical support for solving scaling problems during oilfield water injection [27].

## 2. Experimental Section

### 2.1. Materials

The materials used in this study include the following: maleic anhydride (analytical grade, Aladdin Reagent, Shanghai, China); acrylic acid (analytical grade, Aladdin Reagent, Shanghai, China); 2-acrylamido-2-methylpropanesulfonic acid (analytical grade, Aladdin Reagent, Shanghai, China); ammonium persulfate (analytical grade, Sinopharm Chemical Reagent Co., Ltd., Shanghai, China); sodium hypophosphite (analytical grade, Sinopharm Chemical Reagent Co., Ltd., Shanghai, China); sodium hydroxide (analytical grade, Sinopharm Chemical Reagent Co., Ltd., Shanghai, China); anhydrous ethanol (analytical grade, Sinopharm Chemical Reagent Co., Ltd., Shanghai, China); acetone (analytical grade, Sinopharm Chemical Reagent Co., Ltd., Shanghai, China); deionized water (laboratory-made).

### 2.2. Preparation of the Scale Inhibitor

**Figure 1:**
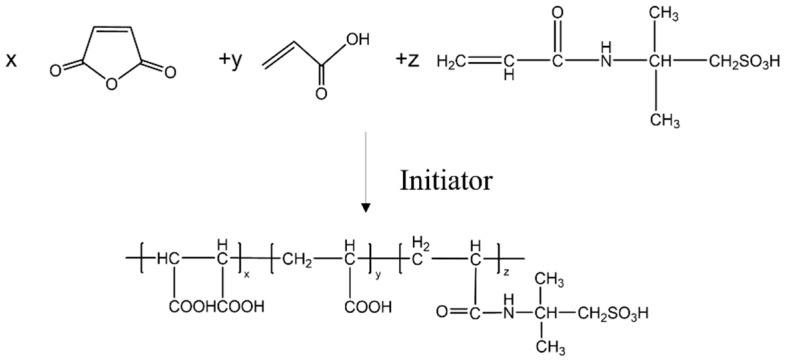
Chemical reaction formula for preparation of scale inhibitor.

Approximately 0.05 mol of maleic anhydride (MA) was dissolved in 50 g of deionized water, then the mixture was added to a four-neck flask and heated. When the temperature was raised to 50 °C, a 20% sodium hydroxide solution was added dropwise to adjust the pH of the system to 5. After stirring for 30 min, 0.18 mol acrylic acid (AA) and 0.03 mol sodium hypophosphite were added, and the temperature was increased to 85 °C. Subsequently, 10% aqueous solution of 2-acrylamido-2-methylpropanesulfonic (AMPS) acid (0.6 mol) and 10% aqueous solution of sodium phosphate (0.3 mol) were added dropwise within 1 h, and the reaction was carried out at 90 °C for 3 h. After cooling, the terpolymer scale inhibitor was obtained and named MA-AA-AMPS (Figure 1).

To further remove impurities (e.g., MA, sodium phosphate, etc.) contained in the scale inhibitor, a mixed solution of absolute ethanol and acetone was used to purify the copolymer product, with the volume ratio of absolute ethanol to acetone set at 3:1. The product was washed with this mixed solution more than 5 times, and the washed product was then vacuum-dried at 50 °C. Through these steps, the purified copolymer scale inhibitor was obtained, with a yield of 91.3%.

### 2.3. Characterization of the Scale Inhibitor

MA-AA-AMPS was ground to 200 mesh and dried, then mixed with potassium bromide powder by the KBr pellet method, ground uniformly, and pressed into pellets. The functional group structure was determined using a Nicolet IS10 (Thermo Fisher, Waltham, MA, USA) Fourier transform infrared spectrometer with a scanning range of 4000~400 cm^−1^. The molecular structure of MA-AA-AMPS was analyzed by proton nuclear magnetic resonance (^1^H NMR, GmbH 400MHz, Bruker BioSpin, Billerica, MA, USA). For thermogravimetric analysis, 5 mg of MA-AA-AMPS (ground to 200 mesh) was tested using a TG 209 F3 (Selb, Germany) Tarsus thermogravimetric analyzer to evaluate its thermal stability, with a temperature range of 20~600 °C and a heating rate of 2 °C/min. Its microstructure was observed using a scanning electron microscope (SEM, Hitachi SU8220, Tokyo, Japan) to clarify its morphological characteristics.

### 2.4. Comprehensive Performance Evaluation of Scale Inhibitor

(1) Determination of scale inhibition rate

The scale inhibition rate was determined by measuring the calcium ion concentration before and after adding the inhibitor using ethylenediamine tetraacetic acid (EDTA) titration with calconcarboxylic acid as an indicator. The experimental parameters were set as follows: Ca^2+^ concentration 0.24 mg·mL^−1^, HCO_3_^−^ concentration 0.732 mg·mL^−1^, pH 9.0, and heating in a constant-temperature water bath at 80 °C for 10 h. The scale inhibition rate (*η*) was calculated using the following formula:

(1) $$\eta =\frac{\rho_{1}-\rho_{0}}{0.24-\rho_{0}}\times 100\%$$

where *η* is the scale inhibition rate (%), *ρ*_1_ is the calcium ion concentration in the solution with inhibitor (mg·mL^−1^), and *ρ*_0_ is the calcium ion concentration in the blank solution without inhibitor (mg·mL^−1^).

For calcium sulfate scale, the same method was used with the following parameters: Ca^2+^ 1500 mg/L, SO_4_^2−^ 3600 mg/L, pH 7.0, 80 °C for 10 h.

(2) Biodegradability assessment

The biodegradability was evaluated by the ratio of BOD_5_ (biochemical oxygen demand over 5 days) to COD (chemical oxygen demand), classified as shown in Table 1 [28].

COD test procedure: Excess potassium dichromate was titrated with ammonium ferrous sulfate using ferroin indicator. The COD value was calculated based on the consumption of the standard solution. The degradation rate was calculated using the following formula:

(2) $$\mathrm{Degradation}\;\mathrm{rate}\;\mathrm{on}\;\mathrm{the}\;n\text{-}\mathrm{th}\;\mathrm{day}=\frac{\mathrm{Initial}\;\mathrm{net}\;\mathrm{COD}\text{-}\mathrm{Net}\;\mathrm{COD}\;\mathrm{on}\;\mathrm{the}\;n\text{-}\mathrm{th}\;\mathrm{day}}{\mathrm{Initial}\;\mathrm{net}\;\mathrm{COD}}$$

## 3. Results and Discussion

### 3.1. Scale Inhibitor Structure Analysis

**Figure 2:**
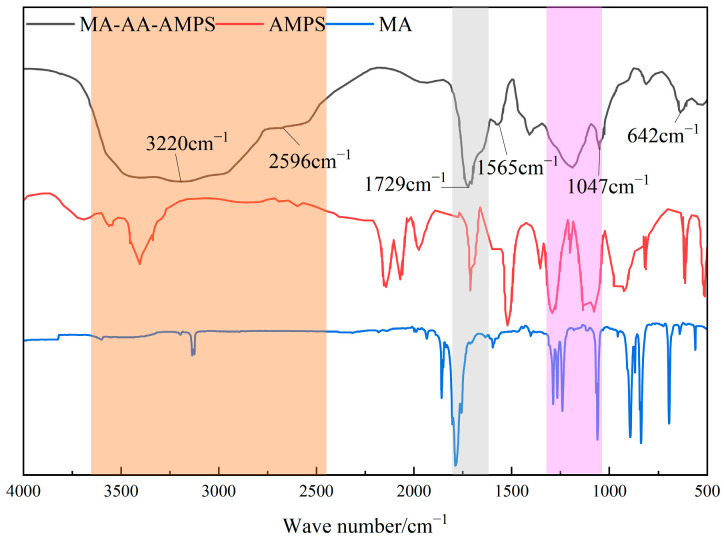
Infrared spectrum of biodegradable scale inhibitor MA-AA-AMPS.

The degradable scale inhibitor MA-AA-AMPS was structurally characterized by infrared spectroscopy. The analysis, combined with the infrared features of monomers MA and AMPS, is as follows (Figure 2). In the characteristic region of C=C double bond (around 1640 cm^−1^ (grey area)), no obvious absorption peak appeared in the spectrum of MA-AA-AMPS, indicating that the reactive monomers had been effectively removed through purification, and the C=C double bonds of the monomers were opened to participate in the polymerization reaction, confirming that the product was a polymer of monomers, such as MA and AMPS. A stretching vibration peak of -OH was observed around 3417 cm^−1^, a characteristic absorption peak of C=O double bond existed at 1729 cm^−1^ (grey area), and a stretching vibration peak of C-O bond appeared at 1412 cm^−1^ [27]. The broad peaks in the range of 2596–3220 cm^−1^ (orange area) jointly confirmed the presence of carboxylic acid groups (-COOH) in the product, which was consistent with the reaction path of MA ring, opening hydrolysis to generate carboxylic acid groups. The absorption peak at 1565 cm^−1^ (grey area) was attributed to the bending vibration of -NH bond in the amide group (-CONH-), proving that the amide functional group of the AMPS monomer was introduced into the product. The stretching vibration peak of the S=O bond at 1047 cm^−1^ (pink area) and the stretching vibration peak of the S-O bond at 642 cm^−1^ were typical infrared characteristics of sulfonic acid groups (-SO_3_H), indicating that the sulfonic acid structure of the AMPS monomer was retained in the product [23]. In conclusion, infrared spectroscopy effectively characterized the functional group composition of MA-AA-AMPS. The polymerization reaction made the C=C double bonds of the monomers disappear, and the product retained carboxylic acid groups derived from MA and amide and sulfonic acid groups derived from AMPS, which was consistent with the structural design of the target copolymer and the requirements for scale inhibition function, providing a structural basis for the study of its scale inhibition performance.

**Figure 3:**
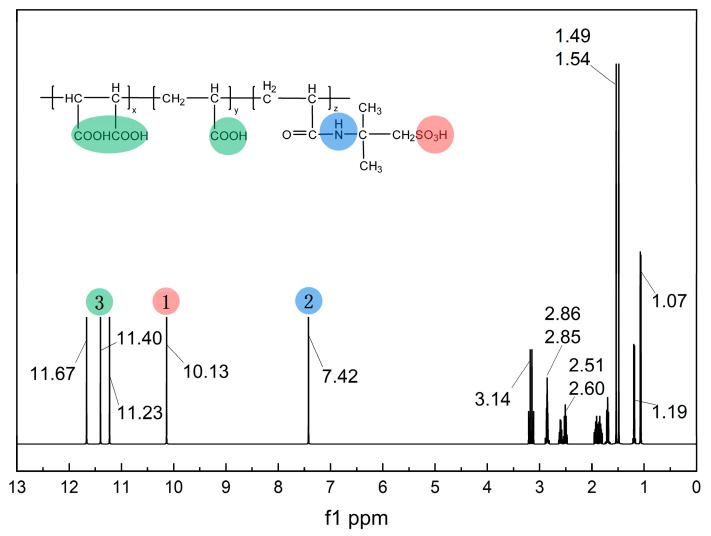
^1^H NMR spectrum of MA-AA-AMPS.

To further verify the successful synthesis of MA-AA-AMPS, a ^1^H NMR analysis was performed (Figure 3). In the low-field region (δ 7.42–11.67), the singlet at δ 10.13–11.67 can be assigned to the active hydrogen signals of carboxyl groups (-COOH) and those adjacent to nitrogen-containing carbonyl groups [27]. These hydrogens exhibit significantly reduced electron density due to the strong electron-withdrawing effect of O and N atoms, resulting in low-field chemical shifts. Moreover, the rapid exchange of active hydrogens weakens the coupling effect, thus showing a singlet feature, which matches the -COOH and nitrogen-containing functional groups (e.g., amide structures) in the molecular structure. The singlet at δ 7.42 can be further assigned to hydrogens on nitrogen-containing heterocycles or amide groups, localized in this region due to the synergistic effect of N electronegativity and carbonyl groups. In the mid-field region (δ 2.00–4.00), the multiplets (e.g., d peaks, m peaks) near δ 2.51–3.14 correspond to hydrogens of methylene (-CH_2_-) and methine (-CH-) groups connected to heteroatoms (N, S, etc.) or electron-withdrawing groups (-SO_3_H, -COOH, etc.) [23]. In the molecule, -SO_3_H and nitrogen-containing side chains reduce the electron density of hydrogens on the connected carbons, shifting the chemical shifts to the mid-field. The spin-spin coupling of adjacent hydrogens (e.g., J coupling for d peaks, multiple couplings for m peaks) complicates the peak shapes, which is consistent with the carbon-hydrogen environments of side chains and functional group connections. In the high-field region (δ 1.00–2.00), the peak cluster at δ 1.07–1.54 is assigned to saturated C-H hydrogens on alkyl chains (e.g., -CH_2_-, -CH_3_ in the molecular backbone). Alkyl hydrogens have high electron density, leading to high-field chemical shifts. Methyl groups (-CH_3_) often show singlets (e.g., the singlet at δ 1.49) due to a relatively uniform surrounding environment. Methylene groups (-CH_2_-) in long-chain alkyls exhibit multiplets due to coupling with adjacent hydrogens, consistent with the carbon-hydrogen distribution in the molecular backbone. In summary, the chemical shifts and peak shapes in the ^1^H NMR spectrum match the electron densities and coupling characteristics of hydrogens in different environments (active hydrogens, hydrogens connected to heteroatoms, alkyl hydrogens) in the MA-AA-AMPS molecular structure. This effectively confirms the presence of functional groups, such as carboxyl, nitrogen-containing carbonyl, and sulfonic acid groups, as well as the connection mode of alkyl chains, verifying the successful synthesis of the MA-AA-AMPS scale inhibitor.

**Figure 4:**
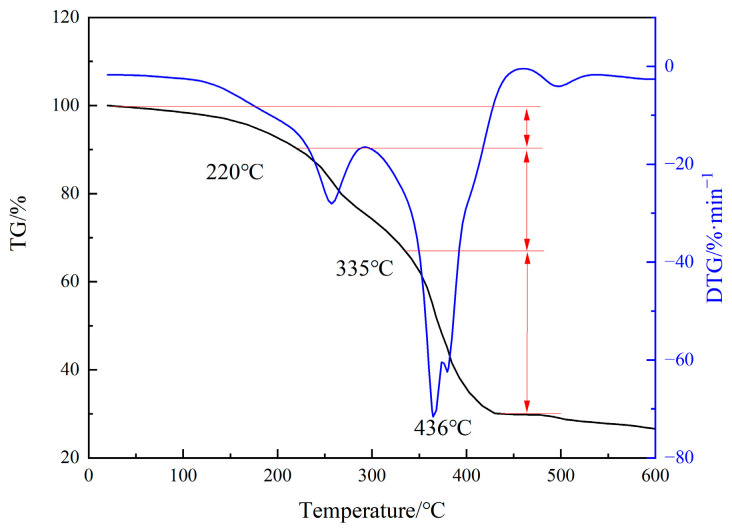
Thermogravimetric curve of degradable scale inhibitor.

Thermogravimetric analysis was performed to obtain the mass loss of the MA-AA-AMPS scale inhibitor at different temperature ranges, enabling quantitative evaluation of its thermal stability (Figure 4). The results show that the thermal decomposition of MA-AA-AMPS mainly occurs in three stages: the first stage (20~220 °C), with a mass loss rate of approximately 10%, which is mainly due to the evaporation of water molecules adsorbed on the polymer; the second stage (220~335 °C), with a mass loss of about 23%, resulting from the decomposition of polymer side chains (e.g., sulfonic acid groups, carboxylic acid groups); and the third stage (335~436 °C), with a mass loss rate of around 37%, primarily caused by the thermal decomposition of the molecular main chain and cleavage of C-C bonds. When the temperature exceeds 436 °C, the scale inhibitor is completely carbonized, leading to minimal mass change. The mass loss at this stage is mainly attributed to the sublimation of carbon residues.

### 3.2. Evaluation of Scale Inhibition Efficiency

**Figure 5:**
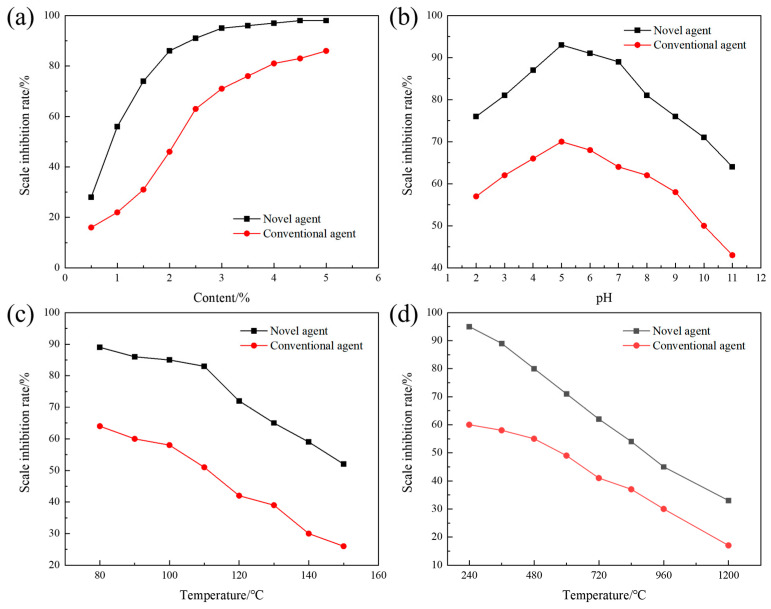
Effects of various variables on the inhibition of calcium carbonate scaling by the ternary polymer MA-AA-AMPS: (**a**) concentration of the scale inhibitor, (**b**) pH, (**c**) temperature, (**d**) calcium ion concentration.

The terpolymer MA-AA-AMPS was successfully synthesized in this study. To investigate the effects of various factors on its efficacy in inhibiting calcium carbonate scaling, static experiments were conducted to evaluate its scale inhibition performance. The terpolymer synthesized under optimal conditions was selected for subsequent assessments, with parameters such as copolymer dosage, water bath temperature, pH value, and calcium ion concentration adjusted individually. As shown in Figure 5a, MA-AA-AMPS exhibits a “low dosage-high efficiency” characteristic: the scale inhibition rate exceeds 80% at a dosage of 2%, which is approximately 2% lower than that of commonly used phosphoric acid scale inhibitors (requiring 4% dosage to achieve similar efficacy). This is attributed to the synergistic effect of carboxylic acid, amide, and sulfonic acid groups in its molecular structure: carboxylic acid groups reduce supersaturation by chelating Ca^2+^, amide groups adsorb onto crystal surfaces via hydrogen bonds, and sulfonic acid groups induce lattice distortion [29]. The combination of these multiple mechanisms enables the scale inhibitor to efficiently occupy crystal growth sites. In contrast, phosphoric acid scale inhibitors rely on single P-O bond chelation, and their scale inhibition rate only tends to stabilize when the dosage is increased to 3%, indicating that a small amount of MA-AA-AMPS can achieve high-efficiency scale inhibition.

With the scale inhibitor concentration set at 3%, the effect of different pH values on the scale inhibition rate of MA-AA-AMPS was tested and compared with that of phosphoric acid scale inhibitors (Figure 5b). The results show that the scale inhibition rate of MA-AA-AMPS is consistently higher than that of phosphoric acid scale inhibitors under all pH conditions, maintaining over 80% in the pH range of 3–8, indicating a wide application range. At low pH (2–5), the molecular chains of MA-AA-AMPS are curled up due to hydrogen bonding. As the pH increases, carboxyl and sulfonic acid groups undergo deprotonation, and enhanced electrostatic repulsion stretches the molecular chains, exposing more active sites (e.g., -COO^−^, -SO_3_^−^) to inhibit calcium carbonate crystal growth through the “adsorption-encapsulation” dual effect. At high pH (>5), the ionization equilibrium of HCO_3_^−^ shifts rightward, leading to a surge in CO_3_^2−^ concentration, which accelerates calcium carbonate nucleation. However, MA-AA-AMPS can still delay crystal aggregation and crystallization through hydrogen bonding between amide groups and crystal surfaces, with a scale inhibition rate (64%) significantly higher than that of phosphoric acid scale inhibitors (43%), breaking the limitation of traditional scale inhibitors with sharply reduced performance under extreme pH conditions. As shown in Figure 5c, temperature significantly affects the scale inhibition effect. In the high-temperature range of 80–150 °C, the scale inhibition rate of MA-AA-AMPS is consistently superior to that of phosphoric acid scale inhibitors, with a fluctuation of <5% before 110 °C. This is because a moderate temperature increase promotes the ionization of side chain groups (e.g., -COOH→-COO^−^), enhancing chelation efficiency with Ca^2+^. When the temperature exceeds 110 °C, although the scale inhibition rate decreases rapidly (still >50% at 150 °C), the thermal stability of C-C bonds and amide bonds in its molecular main chain is better; only partial side chains break, and the remaining carboxylic acid and sulfonic acid groups can still interfere with crystal growth through “point adsorption”, while the scale inhibition rate of phosphoric acid scale inhibitors is <26% at 150 °C. Regarding calcium ion concentration, as the Ca^2+^ concentration increases from 240 mg/L to 1200 mg/L, the scale inhibition rate of MA-AA-AMPS shows a “slow decline-rapid decline” trend (Figure 5d). At a Ca^2+^ concentration of 840 mg/L, the scale inhibition rate remains at 54% because multiple chelating functional groups in the molecule can form stable five-membered/six-membered ring complexes with Ca^2+^, delaying precipitation formation. When the concentration exceeds 840 mg/L, chelating sites become saturated, and excess Ca^2+^ rapidly combines with CO_3_^2−^ to form scale, leading to a sharp drop in the scale inhibition rate (down to 33% at 1200 mg/L). Compared with phosphoric acid scale inhibitors (with a scale inhibition rate of <50% at a Ca^2+^ concentration of 600 mg/L), the chelating capacity of MA-AA-AMPS is increased by approximately 15%. The spatial distribution of its multiple functional groups optimizes the synergistic chelation efficiency for Ca^2+^, expanding the application boundary in high-calcium hard water.

**Figure 6:**
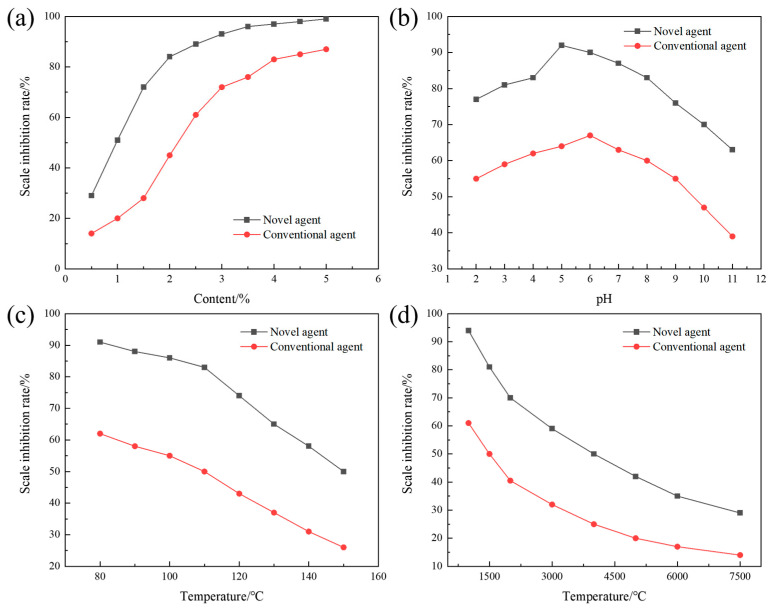
Effects of various variables on the inhibition of calcium sulfate scaling by the ternary polymer MA-AA-AMPS: (**a**) concentration of the scale inhibitor, (**b**) pH, (**c**) temperature, (**d**) calcium ion concentration.

To investigate the effects of various factors on the efficacy of terpolymer MA-AA-AMPS in inhibiting calcium sulfate scaling, static experiments were conducted to evaluate its scale inhibition performance. The terpolymer synthesized under optimal conditions was selected for subsequent assessments, with parameters such as copolymer dosage, water bath temperature, pH value, and calcium ion concentration adjusted individually. As shown in Figure 6a, the scale inhibition rate of MA-AA-AMPS exceeds 80% at a dosage of 2%, while commonly used phosphoric acid scale inhibitors require a 4% dosage to achieve the same effect. This is attributed to the synergistic effect of carboxylic acid, amide, and sulfonic acid groups in its molecular structure: carboxylic acid groups reduce the supersaturation of calcium sulfate by chelating Ca^2+^, inhibiting crystal nucleation. Amide groups adsorb onto the surface of calcium sulfate crystals via hydrogen bonds, blocking growth sites. Sulfonic acid groups induce lattice distortion, disrupting the crystal structure [29]. The combination of these multiple mechanisms enables MA-AA-AMPS to efficiently occupy key growth sites. In contrast, phosphoric acid scale inhibitors rely on single P-O bond chelation, and their scale inhibition rate only tends to stabilize when the dosage is increased to approximately 3%, highlighting the advantage of MA-AA-AMPS in achieving high-efficiency scale inhibition with low dosage.

With the scale inhibitor concentration set at 3%, the effect of different pH values on the scale inhibition rate of MA-AA-AMPS against calcium sulfate was tested and compared with that of phosphoric acid scale inhibitors (Figure 6b). The results show that the scale inhibition rate of MA-AA-AMPS is consistently higher than that of phosphoric acid scale inhibitors under all pH conditions, maintaining over 80% in the pH range of 3–8, indicating a wide application range. At low pH (2–5), the molecular chains of MA-AA-AMPS are curled up due to hydrogen bonding. As pH increases, carboxyl and sulfonic acid groups undergo deprotonation, and enhanced electrostatic repulsion stretches the molecular chains, exposing more active sites, such as -COO^−^ and -SO_3_^−^, to inhibit the crystallization and growth of calcium sulfate crystals through the “adsorption-encapsulation” dual effect. At pH > 5, although changes in the chemical environment of the solution do not significantly alter the scaling driving force of calcium sulfate (unlike calcium carbonate, which is affected by carbonate ionization), MA-AA-AMPS can still delay crystal aggregation and crystallization through hydrogen bonding between amide groups and crystal surfaces, with a scale inhibition rate (92%) significantly higher than that of phosphoric acid scale inhibitors (64%), breaking the limitation of traditional scale inhibitors with sharply reduced performance in acidic/alkaline water. In the high-temperature range of 80–150 °C, the scale inhibition rate of MA-AA-AMPS against calcium sulfate is consistently superior to that of phosphoric acid scale inhibitors (Figure 6c). The scale inhibition rate of MA-AA-AMPS fluctuates by <5% before 110 °C because the moderate temperature increase promotes the ionization of side chain groups (e.g., -COOH→-COO^−^), enhancing chelation efficiency with Ca^2+^ and more effectively inhibiting the nucleation and growth of calcium sulfate crystals. When the temperature exceeds 110 °C, although the scale inhibition rate decreases rapidly, it remains >50% at 150 °C. Compared with phosphoric acid scale inhibitors (with a scale inhibition rate <26% at 150 °C), the thermal stability of C-C bonds and amide bonds in the molecular main chain of MA-AA-AMPS is better; only partial side chains break, and the remaining carboxylic acid and sulfonic acid groups can interfere with the growth of calcium sulfate crystals through “point adsorption”, exhibiting good temperature resistance and solving the problem of high-temperature failure of traditional scale inhibitors.

As shown in Figure 6d, as the Ca^2+^ concentration increases from 1500 mg/L to 7500 mg/L, the scale inhibition rate of MA-AA-AMPS against calcium sulfate shows a “rapid decline-slow decline” trend. At a Ca^2+^ concentration of 4000 mg/L, the scale inhibition rate remains at 50% because multiple chelating functional groups in the molecule can form stable five-membered/six-membered ring complexes with Ca^2+^, binding Ca^2+^ and delaying the formation of calcium sulfate precipitation. When the concentration exceeds 2000 mg/L, chelating sites become saturated, and excess Ca^2+^ rapidly combines with SO_4_^2−^ to form scale, leading to a sharp drop in the scale inhibition rate (down to 29% at 7500 mg/L). Compared with phosphoric acid scale inhibitors (with a scale inhibition rate <14% at 7500 mg/L), the chelating capacity of MA-AA-AMPS is increased by approximately 15%. The spatial distribution of its multiple functional groups optimizes the synergistic chelation efficiency for Ca^2+^, expanding the application boundary in high-calcium hard water.

**Figure 7:**
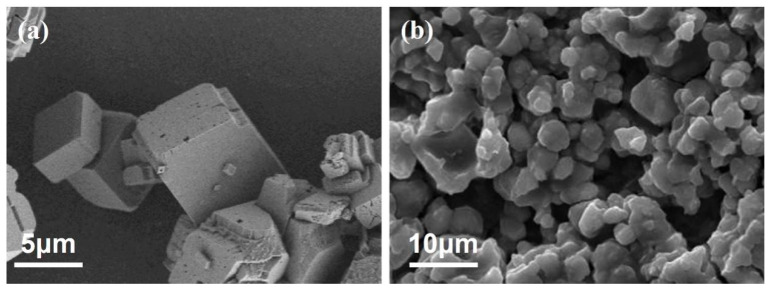
SEM images of calcium carbonate crystals: (**a**) no scale inhibitor is added; (**b**) MA-AA-AMPS scale inhibitor is added.

Calcium carbonate crystalline phases are classified into anhydrous and hydrated phases, with the anhydrous phase further subdivided into three crystal forms: calcite, aragonite, and vaterite. In natural environments, calcite is the most dominant and common form of calcium carbonate due to its excellent thermodynamic stability. Without the addition of scale inhibitors, calcite-phase calcium carbonate crystals exhibit a regular cubic morphology with smooth and flat surfaces, free of obvious pores, flaws, or other defects (Figure 7a). However, after introducing the MA-AA-AMPS scale inhibitor, the surface structure of calcium carbonate crystals undergoes significant changes: the crystals become loose and porous overall, most exhibit irregular scaling characteristics, some even form irregular flocculent spherical morphologies, and the crystal edges show softening (Figure 7b).

Based on the changes in crystal morphology (Figure 7), the inhibition mechanism of MA-AA-AMPS on calcium carbonate scaling is mainly attributed to the synergistic effect of lattice distortion and chelation. Carboxyl and sulfonic acid groups in the copolymer molecules selectively adsorb onto the active surfaces of calcium carbonate crystals, disrupting their original regular structure. This structural damage not only hinders the normal growth of crystals but also makes them more susceptible to being scoured and stripped under water flow. Meanwhile, the chelation of calcium ions by carboxyl groups and the synergistic destruction of calcium carbonate lattices by other groups further enhance the scale inhibition effect, collectively achieving effective inhibition of calcium carbonate scaling.

### 3.3. Scale Inhibition Mechanism

**Figure 8:**
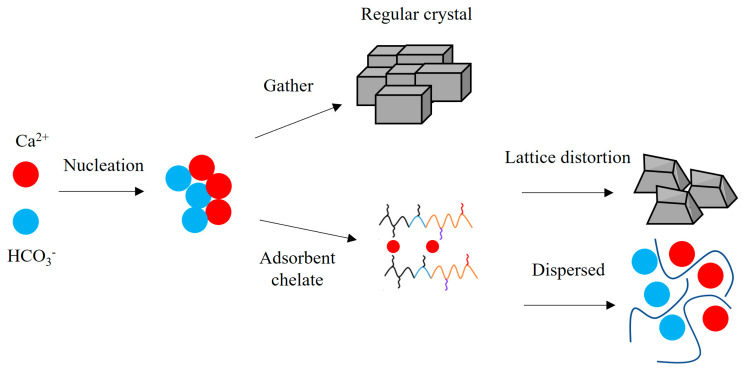
Schematic diagram of action mechanism of scale inhibitor.

Based on the molecular structure characterization and scale inhibition performance test results of the terpolymer MA-AA-AMPS, its scale inhibition mechanism can be analyzed as shown in Figure 8. As illustrated in Figure 8, the side chains of MA-AA-AMPS molecules contain functional groups, such as carboxylic acid, sulfonic acid, and amide groups. Among them, carboxylic acid groups adsorb onto the surfaces of calcium carbonate and calcium sulfate nuclei, increasing the repulsion between different nuclei, which causes distortion during crystal growth and forms layered or porous structures. Secondly, sulfonic acid groups in the molecular chain can enhance the dispersibility and solubility of calcium carbonate and calcium sulfate precipitates. Amide groups interfere with the growth of calcium carbonate and calcium sulfate crystals through adsorption and chelation, leading to the formation of irregular structures. Under the synergistic effect of multiple functional groups, MA-AA-AMPS hinders the normal growth of calcium carbonate crystals and promotes the formation of defective porous structures, thereby effectively inhibiting the generation of calcium carbonate precipitates.

**Figure 9:**
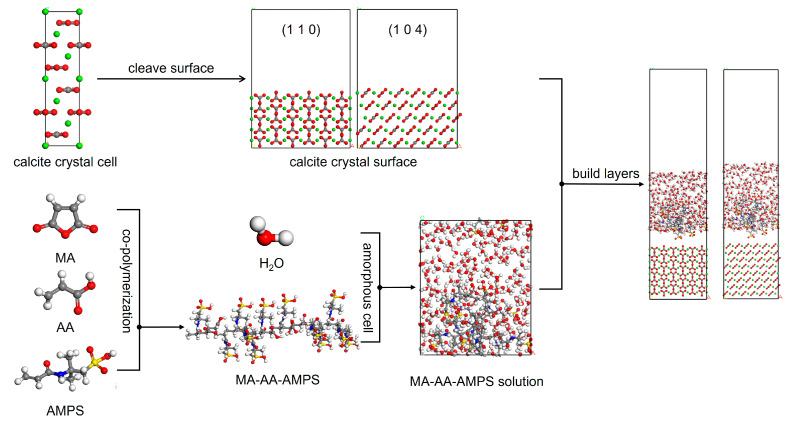
Interface model construction of MA-AA-AMPS copolymer-calcite (1 1 0) (1 0 4) crystal plane (O = red, Ca = green, H = white, C = gray, N = blue).

According to the existing literature, calcite is the most stable polymorph among the three crystal forms of CaCO_3_, with the (1 1 0) and (1 0 4) crystal planes serving as its primary growth faces. The molecular models of the calcite surface and maleic anhydride-acrylic acid-2-acrylamido-2-methylpropanesulfonic acid (MA-AA-AMPS) copolymer were constructed using the Materials Studio 2023 (MS) software package. Subsequent structural optimization via energy minimization and molecular dynamics (MD) simulations were performed [30]. The unit cell of calcite was retrieved from the American Mineralogist Crystal Structure Database (AMCSD), followed by optimization using the COMPASS force field. The optimized calcite crystal was cleaved along the (1 1 0) and (1 0 4) planes, respectively, and then subjected to supercell expansion. This process yielded supercells with dimensions of 24.287427 Å × 19.125326 Å × 32.603196 Å (for the (1 1 0) plane) and 24.287427 Å × 14.970004 Å × 33.738492 Å (for the (1 0 4) plane). To ensure consistency between the simulation results and experimental data, the MA-AA-AMPS copolymer was designed with a degree of polymerization of 17, and the molar ratio of MA:AA:AMPS was set to 1:3.6:12. For investigating the interaction between the MA-AA-AMPS copolymer and the calcite surface in an aqueous environment, a “liquid layer” composed of 200 water molecules and 1 MA-AA-AMPS molecule was prepared. This liquid layer was added to the simulation box and positioned adjacent to the (1 1 0) and (1 0 4) surfaces as the initial state. A vacuum slab with a thickness of 15 Å was introduced along the Z-axis (c-axis) to eliminate the influence of free boundaries on the structure. The interface model is illustrated in Figure 9. On this basis, the Berebdsen temperature control method was adopted, and micro-canonical ensemble -canonical ensemble - micro-canonical ensemble (NVT-NPT-NVT) ensemble simulations were used to simulate the model at 353.15 K for 50 ps-1000 ps-200 ps.

Binding energy indicates the adsorption probability and strength between scale inhibitors and the surface of CaCO_3_ crystals. The formula for calculating the binding energy between MA-AA-AMPS and the (1 1 0) and (1 0 4) planes of CaCO_3_ crystals is as follows:

(3) $$E_{int}=E_{A\text{-}B\text{-}C}-\left(E_{A}+E_{B}+E_{C}\right)$$

Here, *E*_A-B-C_ represents the total binding energy between the MA-AA-AMPS aqueous solution and the CaCO_3_ crystal; *E*_A_ is the total binding energy between MA-AA-AMPS molecules and water; *E*_B_; denotes the total binding energy between the CaCO_3_ crystal and water; *E*_C_ stands for the total binding energy between water molecules; and *E*_int_ is the interaction energy between MA-AA-AMPS molecules and the CaCO_3_ crystal. As observed in Table 2, the *E*_int_; between the MA-AA-AMPS molecules and CaCO_3_ crystals is negative, indicating that the binding of MA-AA-AMPS to CaCO_3_ crystals is exothermic. This confirms that MA-AA-AMPS molecules can stably bind to CaCO_3_, thereby inhibiting the growth of CaCO_3_. Meanwhile, the binding energy *E*_A-B-C_ between the MA-AA-AMPS aqueous solution and CaCO_3_ crystals is much lower than the interaction energy *E*_int_; between the MA-AA-AMPS molecules and CaCO_3_ crystals, suggesting that water molecules can enhance the binding energy between MA-AA-AMPS molecules and CaCO_3_. This further reflects the scale inhibition performance of the MA-AA-AMPS inhibitor, and the conclusion is consistent with the experimental results.

**Figure 10:**
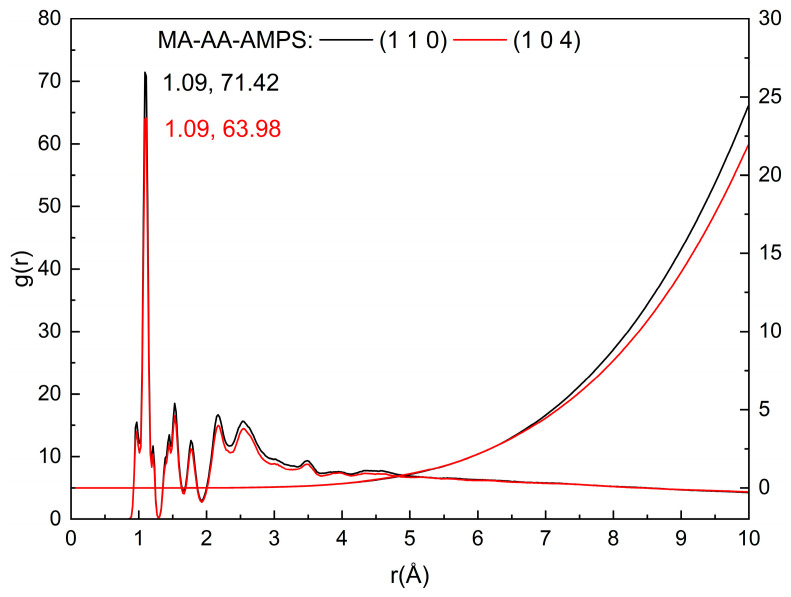
Radial distribution function of O atoms on MA-AA-AMPS and Ca on the calcite (1 1 0) crystal plane.

To further explore the inhibition mechanism of the maleic anhydride-acrylic acid-2-acrylamido-2-methylpropanesulfonic acid (MA-AA-AMPS) copolymer, the radial distribution function (RDF) and coordination number (CN) of MA-AA-AMPS on different calcite crystal planes were analyzed based on molecular dynamics (MD) simulation results (Figure 10) [24]. In general, in the g(r)-r plot, peaks within 3.5 Å are mainly composed of chemical bonds and hydrogen bonds, while peaks beyond 3.5 Å are primarily formed by Coulomb forces and van der Waals forces [31,32]. As shown in Figure 9, the first sharp peak in the g(r) curves of both the (1 0 4) and (1 1 0) planes appears at approximately 1.09 Å. This value is slightly greater than the sum of the covalent radii of oxygen and hydrogen atoms (0.96 Å), indicating the presence of strong hydrogen bonds between the MA-AA-AMPS copolymer and the calcite surface, with the length of these hydrogen bonds being shorter than that of normal hydrogen bonds. A sharp peak is observed at r = 2.53 Å, which matches the Ca-O bond length of 2.39 Å. This phenomenon arises because the carboxyl oxygen atoms of MA-AA-AMPS carry a negative charge, while calcium ions (Ca^2+^) carry a positive charge, leading to the formation of strong ionic bonds between them. Additionally, MA-AA-AMPS exhibits a higher binding affinity for Ca^2+^ on the (1 1 0) plane compared to the (1 0 4) plane. The intensity of the first peak in the g(r) curve of the (1 1 0) surface is greater than that of the (1 0 4) surface, which is consistent with the findings from binding energy studies. Meanwhile, the variation in the CN curve further confirms that the oxygen-containing functional groups (e.g., sulfonic acid groups, carboxyl groups) in MA-AA-AMPS form a coordination complex structure with the Ca^2+^ on the crystal surface.

### 3.4. Degradation Performance Analysis

The biodegradability and degradation rate of the degradable scale inhibitor were evaluated via BOD_5_ and COD tests.

As shown in the test results in Table 3, repeated measurements indicated that the BOD_5_/COD ratio of the new degradable scale inhibitor was consistently greater than 0.3, classifying it as well biodegradable or biodegradable, thus confirming its good biodegradability.

**Figure 11:**
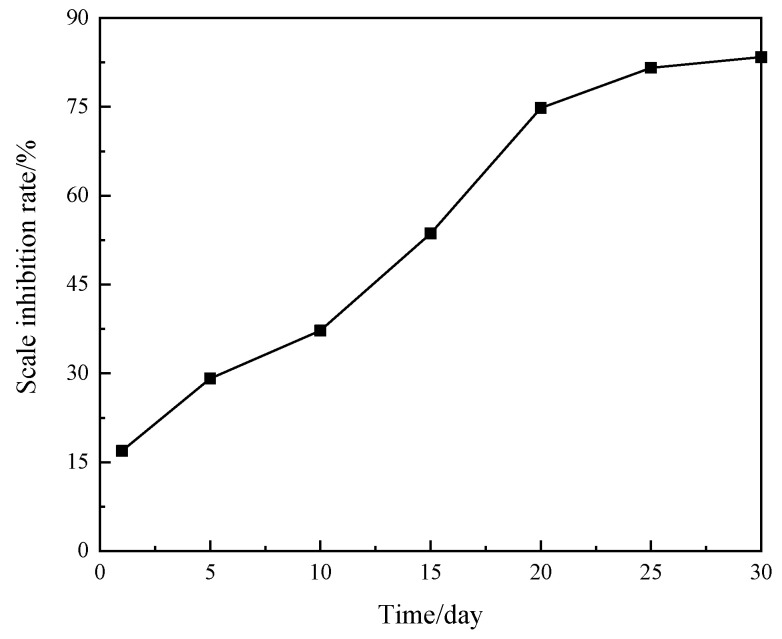
Change of degradation rate of new scale inhibitor with time.

The long-term degradation rate of the new scale inhibitor was analyzed by testing the change in COD variation rate over time. Figure 11 illustrates the effect of degradation time on the degradation rate of the new scale inhibitor. It shows that the degradation rate of the new scale inhibitor increases rapidly with time, reaching equilibrium around 20 days, with a final degradation rate of 83.4% at 30 days. Additionally, the biodegradation rate exceeds 50% after approximately 15 days, demonstrating that the new scale inhibitor exhibits excellent biodegradation performance.

## 4. Conclusions

To address the severe scaling issue in oilfield water injection systems caused by high salt ions and complex environmental factors, this study successfully synthesized a new biodegradable terpolymer scale inhibitor MA-AA-AMPS via aqueous solution polymerization. Systematic characterization and evaluation were conducted on its structural features, scale inhibition performance, thermal stability, and biodegradability, with the main conclusions as follows:

(1) Fourier transform infrared spectroscopy (FTIR) confirmed that MA-AA-AMPS contains carboxylic acid, amide, and sulfonic acid groups, with no residual absorption peak of C=C double bond at 1640 cm^−1^, indicating complete polymerization of the monomers and effective removal through purification. ^1^H NMR further verified the presence of active hydrogens, hydrogens connected to heteroatoms, and alkyl hydrogens in the molecule, consistent with the designed structure of MA-AA-AMPS. Thermogravimetric analysis revealed that its thermal decomposition process is divided into three stages, with the main chain stable up to 436 °C, exhibiting good thermal resistance.

(2) In terms of scale inhibition performance, MA-AA-AMPS showed high-efficiency inhibition on both calcium carbonate and calcium sulfate. For calcium carbonate, it exhibited the advantage of “low dosage-high efficiency”: a dosage of 2% achieved over an 80% scale inhibition rate, reducing the dosage by approximately 2% compared to commonly used phosphoric acid scale inhibitors. It maintained a scale inhibition rate exceeding 80% in the pH range of 3–8 and even reached 64% at pH > 5 (vs. only 43% for phosphoric acid scale inhibitors). It performed better in high-temperature environments of 80–150 °C, with a scale inhibition rate still over 50% at 150 °C. Additionally, it showed good tolerance to calcium ions, maintaining a 54% scale inhibition rate at a calcium ion concentration of 840 mg/L, with its chelating capacity increased by approximately 15% compared to phosphoric acid scale inhibitors. For calcium sulfate, a dosage of 2% achieved a scale inhibition rate over 80% (while phosphoric acid scale inhibitors required 4% dosage). It maintained a scale inhibition rate over 80% in the pH range of 3–8, reaching 92% at pH > 5 (vs. 64% for phosphoric acid scale inhibitors). Its scale inhibition rate remained over 50% at 150 °C and maintained 50% at a calcium ion concentration of 4000 mg/L, showing significantly better adaptability to high calcium levels than traditional scale inhibitors.

(3) The scale inhibition mechanism of MA-AA-AMPS relies on the synergistic effect of functional groups: carboxylic acid and sulfonic acid groups adsorb to disrupt crystal structures, amide groups enhance surface binding, and carboxylic acid groups chelate Ca^2+^ to reduce supersaturation, collectively inhibiting crystal growth and aggregation. Biodegradability tests showed that its BOD_5_/COD ratios were all >0.3, with a 30-day degradation rate of 83.4% and over 50% within 15 days, classifying it as an environmentally friendly scale inhibitor.

## Figures and Tables

**Table 1:** Classification of biodegradation degree.

BOD_5_/COD	Biodegradability
>0.58	Completely biodegradable
0.45–0.58	Well biodegradable
0.3–0.45	Biodegradable
0.1–0.3	Poorly biodegradable
<0.1	Non-biodegradable

BOD_5_ test procedure: A solution of the inhibitor was prepared and placed in a brown bottle with a denitrification inhibitor and NaOH pellets. The bottle was sealed and incubated at 20 °C for 5 days in a biochemical incubator. The BOD_5_ value was measured using a BOD_5_ analyzer.

**Table 2:** The binding energy between MA-AA-AMPS and CaCO_3_ crystal (1 1 0) and (1 0 4) faces.

	*E*_A-B-C_ (kcal/mol)	*E*_A_ (kcal/mol)	*E*_B_ (kcal/mol)	*E*_C_ (kcal/mol)	*E*_int_ (kcal/mol)
(1 1 0)	−61,345.51	−2031.61	−56,581.03	0	−2737.36
(1 0 4)	−60,326.40	−1988.91	−55,710.78	0	−2626.71

**Table 3:** Test results of biodegradation degree of MA-AA-AMPS.

Test No.	BOD_5_/COD	Biodegradability
1	0.53	Well biodegradable
2	0.41	Biodegradable
3	0.47	Well biodegradable
4	0.50	Well biodegradable
5	0.44	Biodegradable

## Data Availability

The original contributions presented in this study are included in the article. Further inquiries can be directed to the corresponding author.
